# Supplementary material for: Uncovering the Relationship between Selenium Status, Age, Health, and Dietary Habits: Insights from a Large Population Study including Nonagenarian Offspring from the MARK-AGE Project
Source: Nutrients. 2023 May 4;15(9):2182. doi: 10.3390/nu15092182 (PMC10180750; doi:10.3390/nu15092182)
Supplement: Supplementary file 1 [file nutrients-15-02182-s001.zip › nutrients-2340789-supplementary.pdf]

Suppl. Table S1 Description of the population under examination

Age Groups	All	1	2	3	4	
**Age range (y)**	**35-75**	**35-44**	**44-54**	**55-64**	**65-75**	
N	3007	483	600	985	939	*P*
Age (y)	57.9 ± 10.7	40.0 ± 2.8	50.2 ± 2.8	60.4 ± 2.8	69.4 ± 3.0	<0.001
Male % (n)	47.7 (1433)	47.6 (230)	46.2 (277)	47.2 (465)	49.1 (461)	0.707
Smoker, never	49.8 (1498)	57.3 (277)	46.0 (276)	46.7 (460)	51.7 (485)	
former	33.3 (1001)	19.7 (95)	31.5 (189)	36.2 (357)	38.4 (360)	<0.001
current	16.9 (508)	23.0 (111)	22.5 (135)	17.1 (168)	9.9 (93)	
Group, GO	17.1 (514)	0.2 (1)	4.2 (25)	24.7 (243)	26.1 (245)	
RASIG	73.2 (2200)	99.6 (481)	92.0 (552)	60.4 (595)	60.9 (572)	<0.001
SGO	9.7 (293)	0.2 (1)	3.8 (23)	14.9 (147)	13.0 (122)	
BMI (kg/m^2)	26.4 ± 4.5	25.0 ± 4.6	25.7 ± 4.3	26.8 ± 4.6	27.1 ± 4.3	<0.001
BMI classes, < 25	42.6 (1282)	57.6 (278)	49.8 (299)	38.5 (379)	34.7 (326)	
25 to < 30	38.9 (1170)	30.2 (146)	35.5 (213)	40.2 (396)	44.2 (415)	<0.001
≥ 30	18.5 (555)	12.2 (59)	14.7 (88)	21.3 (210)	21.1 (198)	
Finland	9.5 (285)	1.9 (9)	4.0 (24)	12.6 (124)	13.6 (128)	
Italy	17.7 (533)	20.7 (100)	18.7 (112)	17.0 (167)	16.4 (154)	
Austria	12.7 (381)	20.3 (98)	16.0 (96)	9.7 (96)	9.7 (91)	
Greece	13.3 (401)	19.3 (93)	17.5 (105)	10.6 (104)	10.5 (99)	<0.001
Poland	16.1 (484)	18.8 (91)	115.3 (92)	17.3 (170)	14.0 (131)	
The Netherlands	7.0 (211)	0.0 (0)	0.5 (3)	8.9 (88)	12.8 (120)	
Belgium	12.2 (366)	6.4 (31)	12.3 (74)	14.1 (139)	13.0 (122)	
Germany	11.5 (346)	12.6 (61)	15.7 (94)	9.8 (97)	10.0 (94)	

[1]Values are mean ± SD for continuous variables and percentage (number) for categorical variables; one missing case for BMI; p-value: one-way-ANOVA (continuous variables) and Chi-square test (prevalence).

Suppl. Table S2 Distribution of GO, SGO and RASIG subjects in relation to age groups and countries

Age groups		35-44			44-54			55-64			65-75		
Experimental Groups		GO	SGO	RASIG	GO	SGO	RASIG	GO	SGO	RASIG	GO	SGO	RASIG
Center	Finland	0 (0)	0 (0)	100 (9)	33.3 (8)	20.8 (5)	45.8(11)	49.2 (61)	22.6(28)	28.2 (35)	53.9 (69)	18.8(24)	27.3(35)
	Italy	1 (1)	1 (1)	98 (98)	7.1 (8)	5.4 (6)	87.5(98)	24(40)	19.2(32)	56.9 (95)	29.2(45)	11(17)	59.7(92)
	Austria	0 (0)	0 (0)	100 (98)	0 (0)	0 (0)	100(96)	0 (0)	0 (0)	100(96)	0(0)	0(0)	100(91)
	Greece	0 (0)	0 (0)	100 (93)	2.9 (3)	1.9 (2)	95.2 (100)	11.5 (12)	1(1)	87.5(91)	6.1(6)	3(3)	90.9(90)
	Poland	0 (0)	0 (0)	100 (93)	5.4 (5)	3.3 (3)	91.3(84)	22.9 (39)	16.5(28)	60.6(103)	20.6(27)	8.4(11)	71(93)
	The Netherlands	0 (0)	0 (0)	0 (0)	33.3 (1)	66.7 (2)	0(0)	54.5 (48)	45.5 (40)	0(0)	55.8 (67)	44.2(53)	0(0)
	Belgium	0 (0)	0 (0)	100 (31)	0 (0)	6.8 (5)	93.2(69)	30.9 (43)	12.9 (18)	56.1 (78)	25.4(31)	11.5(14)	63.1(77)
	Germany	0 (0)	0 (0)	100 (61)	0(0)	0 (0)	100(94)	0 (0)	0 (0)	100 (97)	0(0)	0(0)	100(94)

The number of subjects is indicated in parentheses

Suppl. Table S3. Effect of age, sex, BMI on serum Se in the RASIG population

			**Plasma Se**	
**Variable**		Stat	**Median (IQ)**	**P**
**Age Group**	**35**	**a**	112.25 (110,115)^{c}	<0.025
	**45**	**b**	115.02 (113,117)	
	**55**	**c**	117.51 (115,119)^{a,d}	
	**65**	**d**	113.95 (111,117)^{c}	
**Recruitment Center**	**Finland**	**a**	142.98 (138,148) ^{c,d,e,g,h}	<0.001
	**Italy**	**b**	141.23 (139,143) ^{c,d,e,g,h}	
	**Austria**	**c**	107.25 (105,109) ^{a,b,c,e,h}	
	**Greece**	**d**	107.60 (105,109) ^{a,b,d,e,h}	
	**Poland**	**e**	97.74 (95,101)^{a,b,d,g,h}	
	**The Netherlands**	**f**	.	
	**Belgium**	**g**	122.85 (120,126) ^{a,b,c,d,e,h}	
	**Germany**	**h**	113.0 (111,116)^{a,b,c,d,e,g}	
**Sex**	**F**	**a**	111.44 (109,113)	<0.057
	**M**	**b**	108.83 (107,110)	
**Classes of BMI**	**< 25**	**a**	117.40 (115,119)	<0.001
	**25 to < 30**	**b**	113.70 (111,116) ^{a}	
	**30 and over**	**c**	109.9 (107,113) ^{a}	

Data are shown as median and IQ (interquartile range). Statistical comparison is performed by non parametric tests: Kruskal Wallis test for three or more groups and Mann-Whitney for two groups. Post-hoc for pairwise comparisons in non-parametric tests are based on Dunn procedure. Significant different groups are identified by superscripts indicated in the column "stats".

Suppl. Figure S1. Linear regression between Plasma Se and age in RASIG population

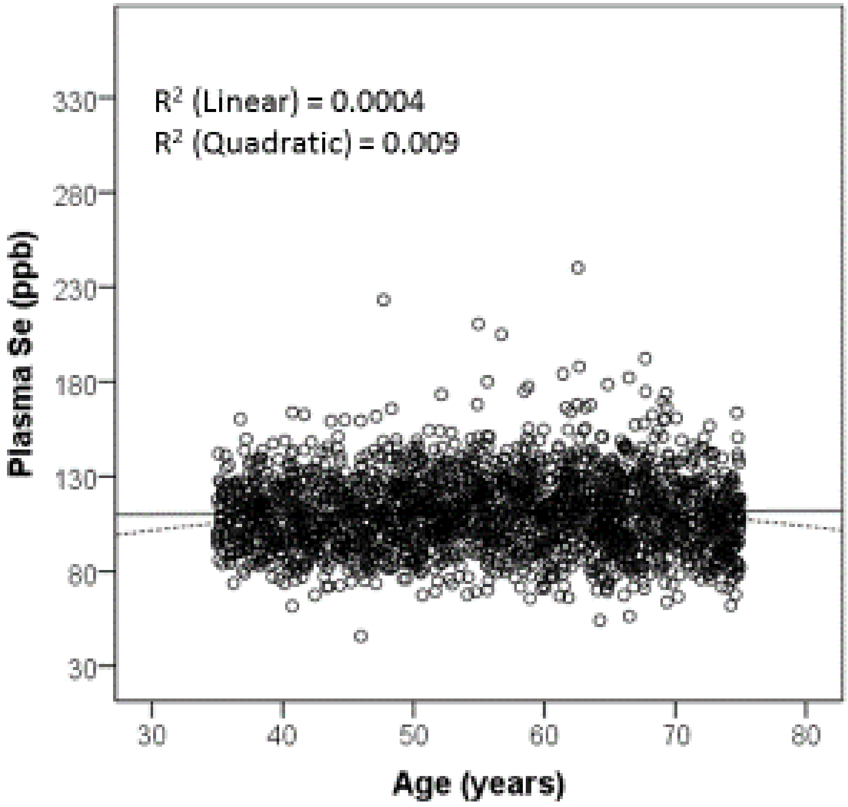

Linear regression between Plasma Se and age (slope 0.003; p=0.878)
Quadratic regression between Plasma Se and age (costant=65.1, b1=1.958, b2=-0.018; p<0.001)

Suppl. Table S4 Association of dietary habits with Plasma Se in Mark-age population

MODEL TERM	COEFFICIENT	SIG	IMPORTANCE
**Vitamin consumption**	-9.71	<0.001	**0.312**
**Fish consumption**	-6.97	<0.001	**0.297**
**Vegetable consumption**	-9.02	<0.001	0.176
**Eggs consumption**	4.58	<0.001	0.133
**Brown bread consumption**	-2,21	0.046	0.038
**Meat consumption**	2.77	0.013	0.027
**Fruit consumption**	-2.23	0.043	0.018

The table shows the most important dietary predictors of plasma Se obtained with automatic regression analyses on Mark-Age population

Suppl. Table S5. Contribution of dietary factors and subject groups (GO, RASIG and SGO) to plasma Se levels by multivariate analysis

Factor	**Dependent Variable**	**Type III Sum of Squares**	**Sig.**
**Recruitment Center**	Plasma Se (ppb)	425600	< 0.001
**Sex**	Plasma Se (ppb)	52	0.694
**Age Classes**	Plasma Se (ppb)	8241	< 0.001
**Subject group (GO, SGO, RASIG)**	Plasma Se (ppb)	360	0.583
**BMI classes**	Plasma Se (ppb)	3100	0.01
**Vitamin consumption**	Plasma Se (ppb)	13317	< 0.001
**Fish consumption**	Plasma Se (ppb)	7360	< 0.001

The analysis included, recruitment center, age and sex as confounding factors

Suppl. Figure S2 Plasma Se levels in MARK-AGE population in relation to vitamin and fish consumption after country stratification

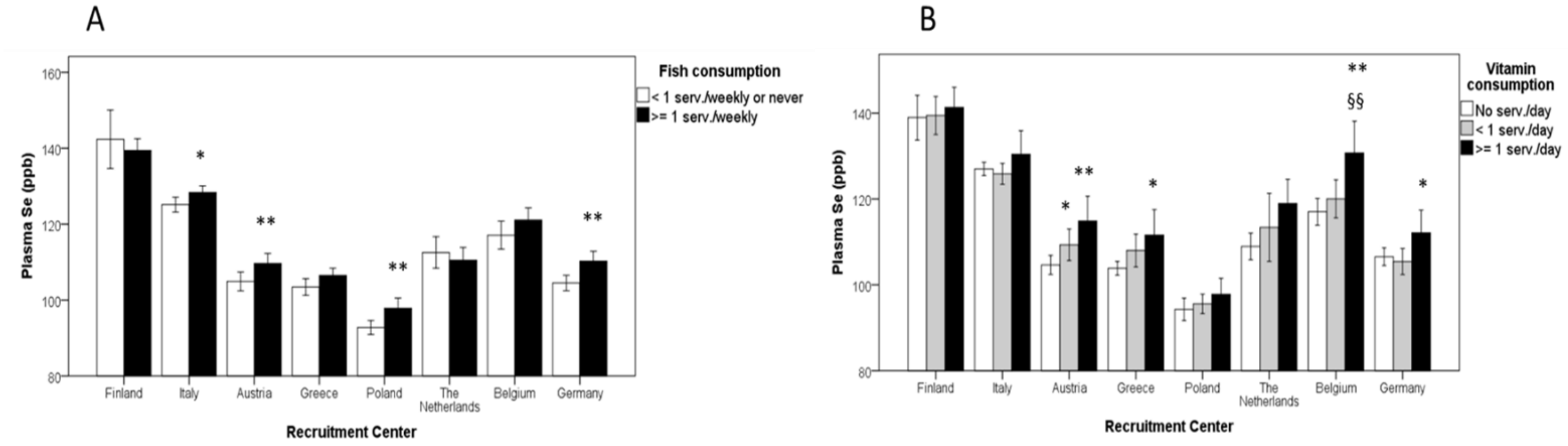

**p<0.01 , *p<0.05 compared to <1 serv./weekly or never (Fig. 2A)

** p<0.01, *p<0.05 compared to no serv./day, §§p<0.05 compared to<1 serv./day (Fig 2B)

Suppl. Figure S3 Se1a and Se1b percentages in RASIG participants stratified by sex

A

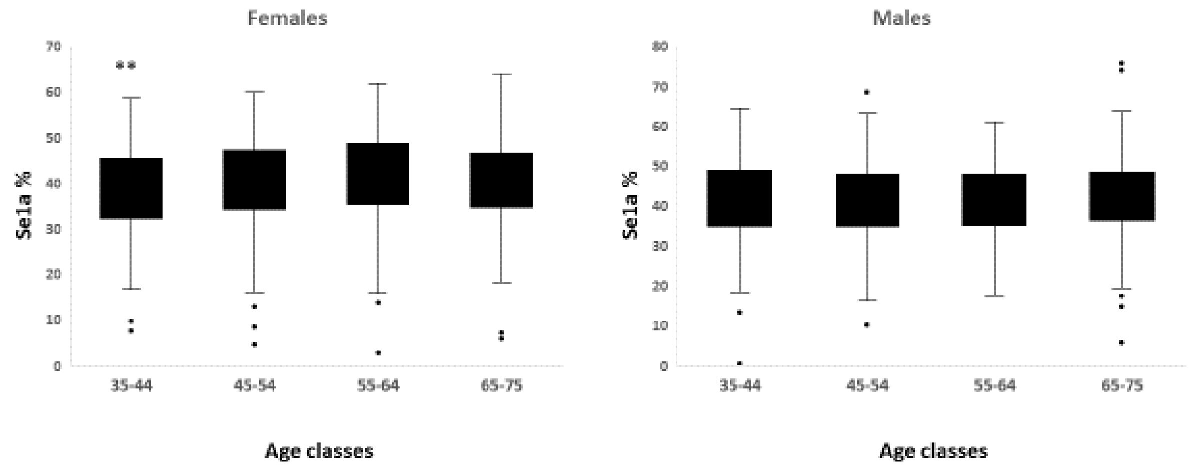

B

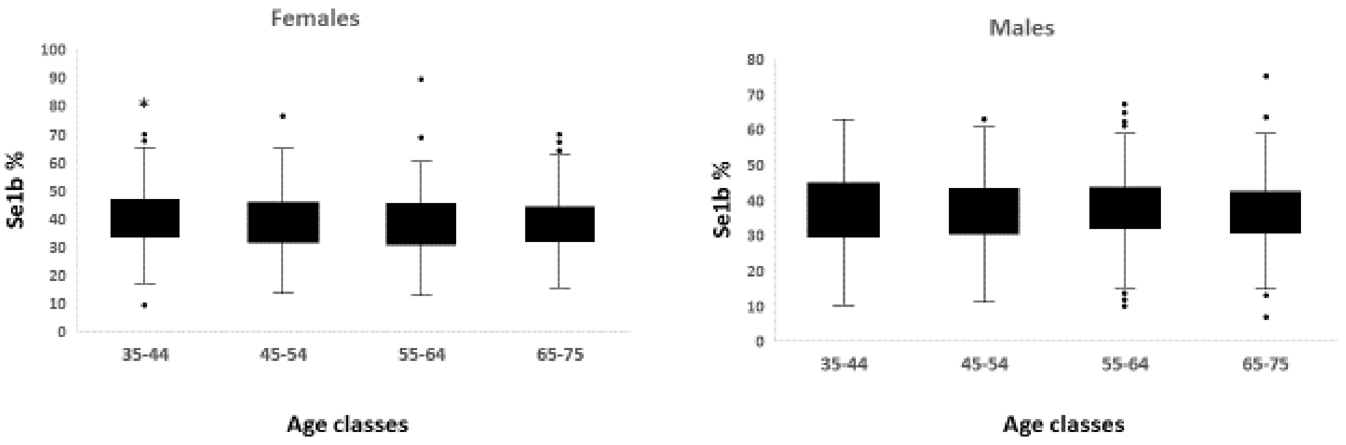

**p<0.01 compared to 45-54 and 55-64 age classes; *p<0.05 compared to 65-75 age class generalized linear model analysis adjusted for BMI and countries

Suppl. Figure S4 Se2 in MAR-AGE population after country stratification

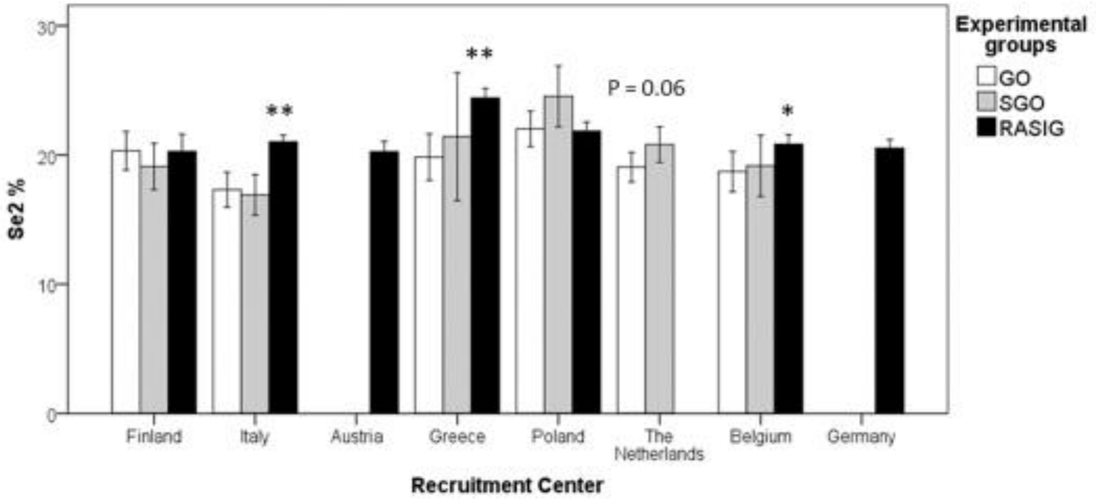

**p<0.001 compared to GO and SGO; *p<0.01 compared to GO

Suppl. Figure S5 Se1a and Se1b percentages in GO, SGO and RASIG participants

A **B**

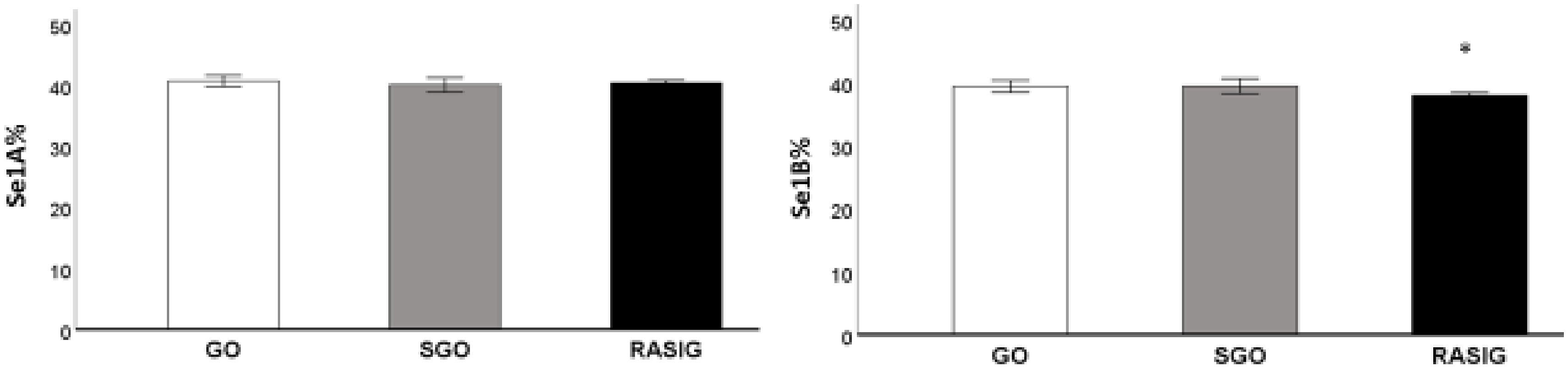

*p<0.05 as compared to GO and SGO Generalized linear mixed model adjusted for adjusting for age, sex, country, BMI fish and vitamin consumption

Suppl. Figure S6. Selenium chromatographic separation

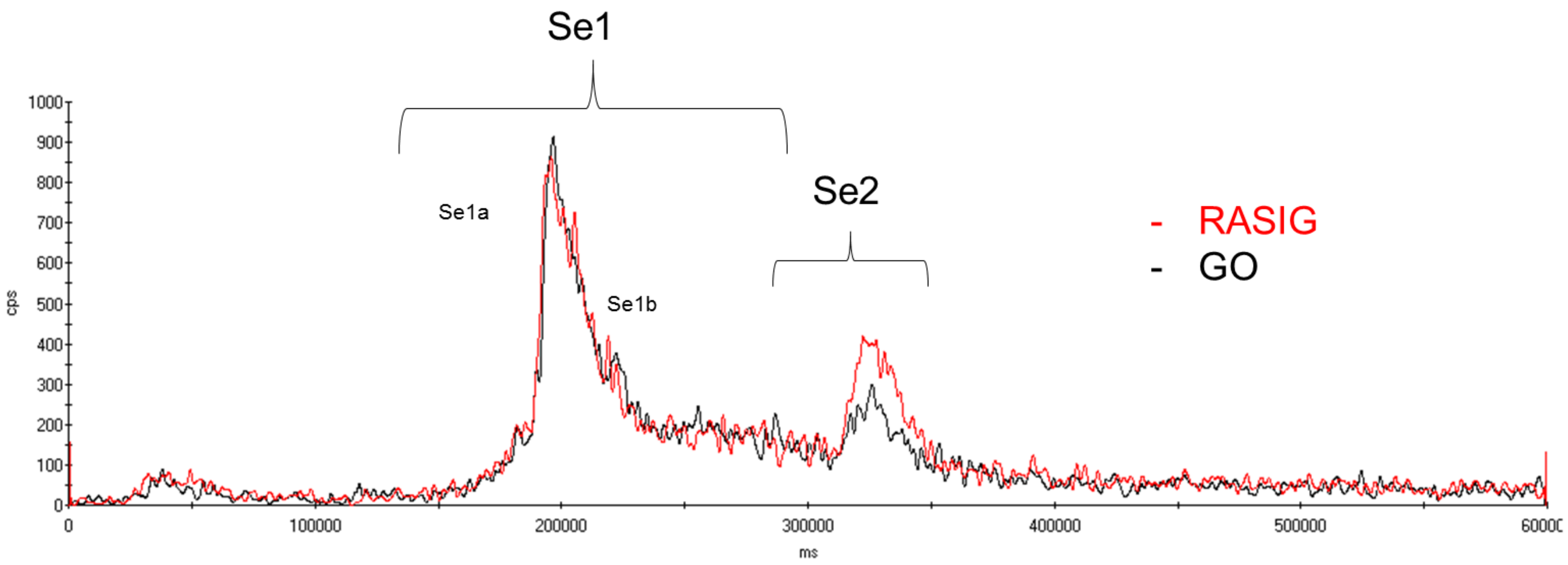

This figure shows a representative comparison between RASIG (red line) and GO (black line) Se chromatographic separations obtained after the injection of 1 ml of human serum into the HPLC-ICP-MS system.

Suppl. Figure S7 Linear regression between Se1a and albumin in RASIG population

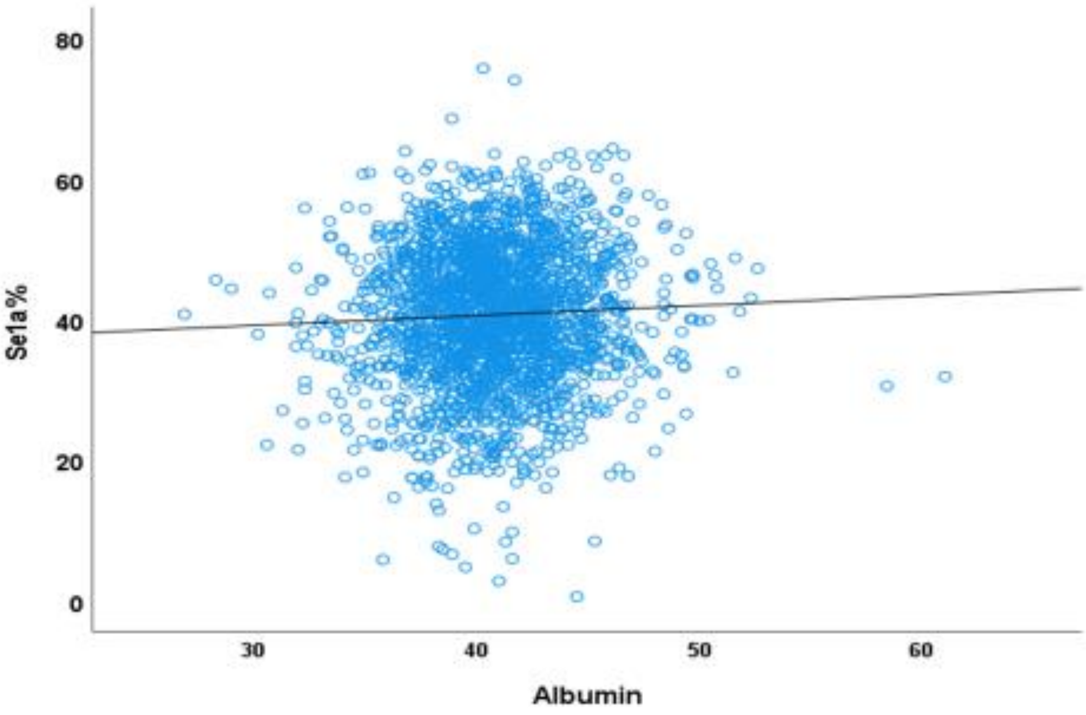

Linear regression between Se1a and albumin after adjusting for age, sex and country (β coefficient 0.05; $p<0.05$)
